# Protocol: A randomized controlled trial to assess effectiveness of a 12-month lifestyle intervention to reduce cardiovascular disease risk in families ten years after pre-eclampsia (FINNCARE)

**DOI:** 10.1016/j.pmedr.2022.101731

**Published:** 2022-02-08

**Authors:** Tiina Jääskeläinen, Anni Kivelä, Michelle Renlund, Seppo Heinonen, Minna Aittasalo, Hannele Laivuori, Taisto Sarkola

**Affiliations:** a Medical and Clinical Genetics, University of Helsinki and Helsinki University Hospital, Helsinki, Finland; b Department of Food and Nutrition, University of Helsinki, Helsinki, Finland; c Children's Hospital, University of Helsinki and Helsinki University Hospital, Helsinki, Finland; d Minerva Foundation Institute for Medical Research, Helsinki, Finland; e Department of Obstetrics and Gynecology, Helsinki University Hospital, Helsinki, Finland; f UKK Institute for Health Promotion Research, Tampere, Finland; g Institute for Molecular Medicine Finland (FIMM), Helsinki Institute of Life Science, University of Helsinki, Helsinki, Finland; h Department of Obstetrics and Gynecology, Tampere University Hospital and Tampere University, Faculty of Medicine and Health Technology, Tampere Center for Child, Adolescent, and Maternal Health Research, Tampere, Finland

**Keywords:** BMI, body mass index, BP, blood pressure, BCW, Behaviour Change Wheel, CVD, cardiovascular disease, DASH, Dietary Approaches to Stop Hypertension, FINNPEC, The Finnish Genetics of Pre-eclampsia Consortium, FFQ, food frequency questionnaire, HRV, heart rate variability, IPAQ, International Physical Activity Questionnaire, PE, pre-eclampsia, Cardiovascular disease, Family, Lifestyle intervention, Pre-eclampsia, Prevention

## Abstract

Pre-eclampsia (PE) is a multisystem progressive disorder affecting 3–5% of pregnancies. PE independently increases the risk for premature cardiovascular disease (CVD) in mothers and their children long-term. The effectiveness of a family-centered lifestyle intervention to lower CVD risk in PE families has not previously been evaluated. In the current FINNCARE study, we prospectively compare CVD risk and CVD progression in PE families (mother, father and child) in a cross-sectional study setting 8–12 years from delivery with non-PE control families of comparable age. Furthermore, we evaluate the effectiveness and feasibility of an interactive web-based behavioral 12-month lifestyle intervention to reduce blood pressure and the CVD risk profile overall in a randomized controlled trial. In total, 300 PE families will be randomized 1:1 to a PE-intervention or a PE-control group, and the 100 non-PE control families similarly followed-up at 12 months. Primary outcome is 24-hour mean systolic BP change from baseline in mother and child. Study aims to provide information on CVD progression and CVD risk management in the family following PE.

## Highlights

• This is appropriately powered 12-month lifestyle intervention for PE families.
• Detailed phenotyping now and during index pregnancy may identify subgroups at CVD risk.
• Genome-wide genotyping is performed for all study participants.
• Process evaluation examines feasibility and follows Medical Research Council guidance.

## 1 Introduction

Pre-eclampsia (PE) is gestational hypertension with new-onset proteinuria and/or organ dysfunction after 20 weeks of gestation (Tranquilli et al., 2014). It is a common cause of maternal and neonatal morbidity and mortality affecting up to 3–5% of all pregnancies overall (Steegers et al., 2010). PE is associated with complications not only during pregnancy as epidemiological studies show increased risk of premature cardiovascular diseases (CVDs) in mothers long-term after delivery (Irgens et al., 2001, Lykke et al., 2009). Systematic reviews demonstrate increased risks of ischemic heart disease and cerebrovascular disease events and overall CVD mortality after PE (Brown et al., 2013, Wu et al., 2017). The increased risk for CVD progression in mothers is related to gestational age at onset and severity of PE (Riise et al., 2017). The risk for CVD, heart failure and stroke are relatively high already during the early 10-year period following delivery (Wu et al., 2017). The association between PE and early CVD progression is not completely understood. The dose–response relationship with the severity of a hypertensive pregnancy disorders and future CVD suggests that the differences in long-term CVD risk may be dependent on the variation of the maternal CVD risk profile (Veerbeek et al., 2015). The burden of common and/or rare genetic variants predisposing to CVD may also vary in women with a history of PE.

Large retrospective registry-based studies show that young adults born to mothers with hypertensive pregnancy disorders have higher blood pressure (BP) and an adverse CVD risk profile compared with offspring of normotensive pregnancies (Alsnes et al., 2017). There are very little long-term data on morbidity and mortality of men who fathered PE pregnancy (Irgens et al., 2001). The PE related CVD risk has been recognized by the American Heart Association (AHA), which recommends that pregnancy history should be included in the evaluation of CVD risk in women (Mosca et al., 2011). Perinatal history of preterm birth and low birth weight – both strongly associated with PE – are together with family history considered important in the diagnostic evaluation of elevated BP in pediatric guidelines (Flynn et al., 2017). However, guidelines addressing postpartum cardiovascular risk assessment after PE show a wide variation (Benschop et al., 2019). Structured follow-up guidelines for CVD prevention in women or children after a PE pregnancy, and evidence-based studies addressing further PE risk stratification, timing and efficacy of interventions to modify CVD risk and progression are lacking. A few ongoing interventions after PE pregnancy are very recently summarized by Jowell et al. (2021). However, these interventions are performed quite soon after pregnancy and do not involve family approach.

It has been demonstrated that major traditional CVD risk factors are modifiable by lifestyle changes and primary lifestyle intervention can help to postpone or even prevent future CVD risk and events in risk populations (Appel et al., 1997). For instance, the Dietary Approaches to Stop Hypertension (DASH) eating pattern is a proven regimen to assist individuals in lowering BP (Sacks et al., 2001). Controlled trials have also shown that implementing a healthy Nordic diet is associated with a reduction in several key CVD risk factors (Uusitupa et al., 2013). Studies on secondary prevention of CVDs have shown that family-centered interventions that include actively involved patients’ partners and other family members are effective due to reinforcement of lifestyle changes involving more than one generation (Pyke et al., 1997, Wood et al., 2008). However, the efficacy of these lifestyle modifications to lower CVD risk in families with a history of PE remains to be determined.

## 2 Study hypothesis and objectives

### 2.1 Hypothesis

We hypothesize that PE is related with CVD progression mediated by BP and CVD risk overall. In addition, abnormalities in BP and the CVD risk profile are modifiable by a 12-month behavioral lifestyle intervention following PE.

### 2.2 Objective

The aim of the FINNCARE study is to assess cardiovascular health and CVD progression in families 8–12 years after a PE pregnancy. Furthermore, we study the feasibility and effectiveness of a 12-month lifestyle intervention to reduce BP and the CVD risk profile overall.

### 2.3 Specific objectives

To prospectively compare CVD risk and CVD progression in PE families (mother, father and child) in a cross-sectional study setting 8–12 years from delivery with non-PE control families (mother, father and child) of comparable age.
To evaluate the effectiveness and feasibility of an interactive web-based behavioral 12-month lifestyle intervention to reduce BP and the CVD risk profile overall in a randomized controlled trial 8–12 years from delivery. PE families will be randomized 1:1 to intervention and control groups. The 12-month difference of change between PE intervention and PE control group will be compared.

## 3 Methods and analysis

### 3.1 Study subjects and design

The study design of FINNCARE includes two parts. First, PE families are compared with non-PE families of comparable age to assess CVD risk and CVD progression in a cross-sectional study setting at 8–12 years from delivery. Second, recruited PE families are randomized 1:1 to PE intervention or PE control groups to assess the feasibility and effectiveness of a 12-month behavioral lifestyle intervention to reduce BP and the CVD risk profile overall.

### 3.2 Study subjects

FINNCARE study subjects are recruited from The Finnish Genetics of Pre-eclampsia Consortium (FINNPEC) multicenter study cohort originally recruited to investigate and search genetic markers predisposing to PE using genome-wide association study (Jääskeläinen et al., 2016). Altogether 1450 nulli- or multiparous women with PE and 1065 women without PE (non-PE) were prospectively recruited during 2008 to 2011 including their partners and newborns. PE was defined as hypertension and proteinuria occurring after 20 weeks of gestation (systolic BP ≥ 140 mmHg and/or diastolic BP ≥ 90 mmHg, and urinary excretion of ≥0.3 g protein in a 24-hour specimen, or 0.3 g/l, or two ≥1 + readings on dipstick) (ACOG, 2002). Each diagnosis was ascertained from hospital records and confirmed independently by a research nurse and a study physician. The samples of all FINNPEC study participants were genome-wide genotyped with Infinium Global Screening Array-24 v2.0 BeadChip (Illumina Inc., San Diego, CA, USA).

**Fig. 1:**
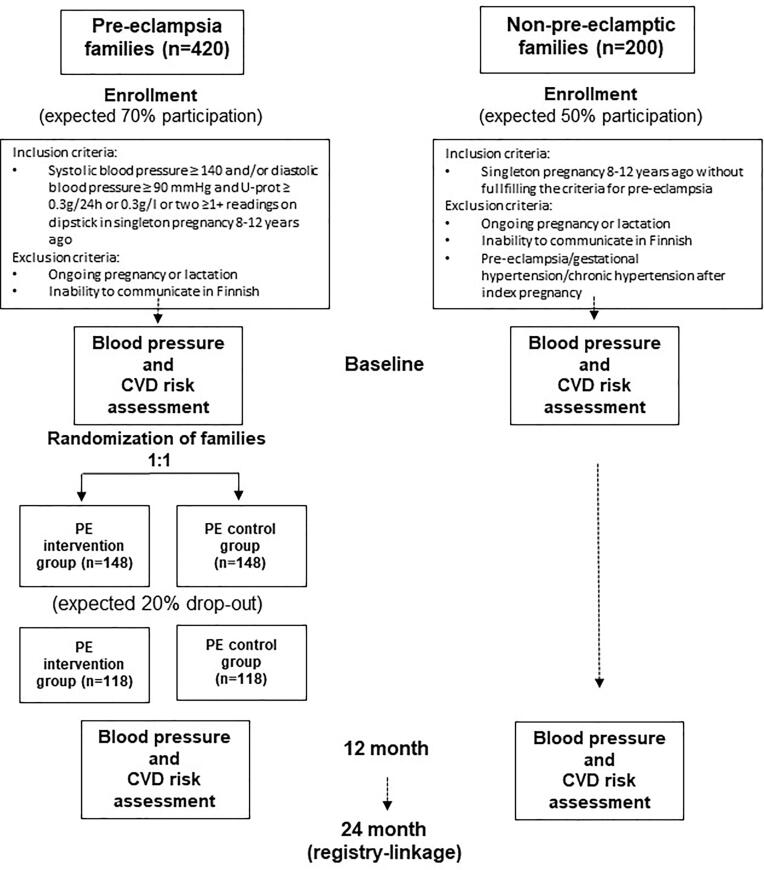
Flowchart of the FINNCARE Study.

PE and non-PE families from the FINNPEC cohort living in the Hospital district of Helsinki and Uusimaa are 8–12 years from delivery randomly contacted by a letter addressed to the mother during 2019–2022 with an offer to without economic compensation participate in the FINNCARE study. In total, 465 PE- and 490 non-PE families were recruited in this particular hospital district during FINNPEC for the prospective study arm in 2008–2011. Based on power calculations and expected participation rate, 420 PE families and 200 non-PE families are contacted (Fig. 1).

Both PE families and non-PE controls represent populations with comprehensive standard primary health care in Finland (including e.g., occupational health care services for mother/father, and child health clinic and school health care services). Exclusion criteria for all mothers include ongoing pregnancy or lactation, multiple pregnancy, and inability to communicate in Finnish. For non-PE families, hospital charts are examined to exclude families with PE/gestational hypertension/chronic hypertension following the index pregnancy. Participation is confirmed with a signed informed consent. All study visits are arranged in a tertiary care setting at the Clinical Trial Unit located at Children's Hospital, Helsinki University Hospital, Finland.

### 3.3 Study sample size, randomization and follow-up

Power calculations were performed in order to detect 5.5 mmHg reduction in maternal systolic BP with a power of 80% and a 2-sided *p*-value of 0.05 for the difference between the groups (PE intervention and PE control) (NICE, 2011/Fig. 5) (NICE Clinical Guidelines, 2011). When expecting 70% participation rate and allowing for a 20% loss to follow-up or missing data, calculations estimate an initial sample size of at least 148 PE women in the intervention group and 148 women in the control group. PE families will be randomized into groups in the recruitment order. The PE control group continues their habitual lifestyle but will perform the same baseline and follow-up measurements as the PE intervention group (Fig. 1). They will be given general written information on healthy eating. Furthermore, 100 non-PE parallel control group that participated in the original FINNPEC study during 2008–2011 will be recruited and assessed at baseline and follow-up.

### 3.4 Lifestyle intervention

Behaviour Change Wheel (BCW) (Michie et al., 2011) was used as a theoretical background in designing the lifestyle intervention. The step-by-step application of BCW (Michie et al., 2014) consists of defining the problem (target behaviours), diagnosing what needs to change in the sources of target behaviours (**C**apability, **O**pportunity, **M**otivation; COM-B model) and identifying intervention functions and behaviour change techniques (BCTs) most likely to bring about change. Finally, the delivery modes were selected with emphasis on affordability, practicability, effectiveness, acceptability, safety and equity – all crucial for the further transferability of the intervention into practice. Both existing (e.g. Smart Family, HulaHula) and new (a web-based portal) delivery modes are combined in the intervention. The content of the 12-month intervention is described in Table 1. Five target behaviours relevant for cardiovascular health are targeted in the intervention:

1) improving the quality of fat in the diet
2) increasing the consumption of foods rich in fiber
3) decreasing the use of salt
4) increasing physical activity
5) reducing smoking.

**Table 1:** Content of the intervention.

Week	Intervention content in relation to target behaviours					Primary behaviour change techniques (BCTs)	Delivery mode
	Diet			Physical activity	Smoking		
	Improving the quality of fat in the diet	Increasing the consumption of foods rich in fiber	Decreasing the use of salt	Increasing leisure time physical activity	Reducing smoking		
1	Assessing current lifestyles to identify and strengthen the positive aspects and to evoke need for change				Telling about the health hazards of smoking and referring to help	Prompt self-monitoring of behaviour; Prompt focus on past success; Goal setting	Face-to-face meeting: Smart family Referral to stumppi.fi
2				Telling about the easiness and benefits of increasing leisure physical activity. Highlighting that all movement that helps stay physically active is important. Introducing a mobile application to help to increase physical activity together as a family.		Provide information on consequences of behaviour in general; Action planning; Helping in setting goals, making weekly plans and reviewing progress.	Web portal: slides, worksheet (screen time), HulaHula application
3	Laboratory tests					Provide information on consequences of behaviour to the individual, Goal setting	Web portal
4–6	Telling about the benefits of healthy eating with emphasis on eating as a source of joy and importance of eating together. Showing how to utilize plate model, food labels and Heart Symbol® in everyday life					Provide information on consequences of behaviour in general; Prompt self-monitoring of behaviour Demonstrate the behaviour; Goal setting; Action planning; Provide feedback on performance	Web portal; slides, videos, worksheets, practical tasks
7				Encouraging families to find new, easy ways to increase physical activity (“living room circus”)		Provide information on consequences of behaviour in general; Prompt self-monitoring of behaviour	Web portal: slides, videos
**8**–11, 16	Showing how **to improve quality of fat (dietary fats, fish and nuts).** Helping in setting behaviour-specific goals, making weekly plans and reviewing progress. Giving feedback on progress.			Discussing the use of mobile application. First badges.		Demonstrate the behaviour; Goal setting; Action planning; Prompt review of behavioural goals; Provide feedback on performance	Web portal: videos, worksheets, slides, templates for action plans and monitoring
11	Discussing progress					Provide feedback on performance	Phone call
12–26	Showing how **to increase foods rich in fiber (fruit, berries and vegetables, whole grains) and limit salt**. Helping in setting behaviour-specific goals, making weekly plans and reviewing progress. Giving feedback on progress.					Demonstrate the behaviour; Goal setting; Action planning; Prompt review of behavioural goals; Provide feedback on performance	Web portal: videos, worksheets, slides, templates for action plans and monitoring
21–22	Discussing progress, regular eating rhythm					Provide feedback on performance; Demonstrate the behaviour; Goal setting; Action planning	Phone call
27				Telling about the benefits of spending time in nature and outdoors.		Demonstrate the behaviour; Demonstrate the behaviour; Goal setting; Action planning;	Web portal; worksheets, bulletin board
28	Discussing about sugar consumption, encouraging families to pay attention to sugar content of food products, especially snacks					Demonstrate the behaviour; Goal setting; Action planning; Provide feedback on performance	Web portal
29–31	Discussing progress					Provide feedback on performance	Phone call
29–31	Showing how to do healthy **grocery shopping.** Helping in identifying and managing situations, where relapses are possible.					Demonstrate the behaviour; Relapse prevention	Web portal; video; Healthy grocery shopping pocket guide
32–46	Highlighting specific things in diet: fruits, berries, vegetables, vegetable protein sources, eggs, breakfast, nuts, unsaturated fats. Proving the chance to keep a three day dietary record					Demonstrate the behaviour; Goal setting; Action planning; Provide feedback on performance and dietary recording	Web portal
46	Discussing progress					Provide feedback on performance	Phone call
47–52	Evaluation: how the goals have been achieved? Succeeds and barrier identification, motivation, future goals					Demonstrate the behaviour; Goal setting; Action planning; Provide feedback on performance, new laboratory results	Web portal
Extra	Providing seasonal tips for e.g. healthy Christmas eating and physical activity. Reinforcing successful performance. Strengthening self-confidence. Showing how to select healthy snacks. Helping in altering the home environment to more supportive for changes, e.g. removing unhealthy snacks to less visible places. Summertime: proving healthy barbecue recipes					Prompt self-monitoring of behaviour; Prompt practice	Web portal, web-pages, recipes, bulletin board

In identifying the sources of behaviour and intervention functions previous literature on women with PE (Hoedjes et al., 2012) and gestational diabetes (Nicklas et al., 2011) was utilized.

At baseline, the PE families in the intervention group have one face-to-face dietary counseling session of 60 min with a nutritionist following measurements and data collection. During the session a family-centered lifestyle counseling method Smart Family (“Neuvokas Perhe” in Finnish) developed by the Finnish Heart Association is utilized (Kyttälä et al., 2014). It is based on motivational interviewing and solution focused counseling. The Smart Family’s self-assessment tool helps the family members to evaluate family’s lifestyle habits, recognize strengths, set goals for improvement and monitor their achievement of the goals, and thus empower the family for a lifestyle change. The intervention continues in an interactive web-based portal which was created for the study in 2019 (HowSpace platform provided by Humap Software Ltd.). Different modules related to target behaviours include assignments, activities, quizzes and related videos (Table 1). Most of them are completed together as a family but there are also material and tasks that are designed only for parents or only for children. The families get individual feedback on assignments and supportive web-based counseling is provided by the nutritionist. The researchers provide families technical support via telephone for using the portal, e-mail reminders about completing the modules and two phone calls to facilitate adherence to the intervention. HulaHula sport and activity mobile pedometer application (provided by Sunday Morning Solutions Ltd.) is used to increase physical activity of the families. It activates and encourages families to increase physical activity through gamification. The web portal does not include a specific module for reducing smoking, but two smoking related questions are integrated into the Smart Family’s self-assessment tool and smoking is discussed during the face-to-face session. If mother and/or father show willingness to reduce smoking, she/he is referred to Stumppi (www.stumppi.fi) service provided by the Organization for Respiratory Health to receive smoking cessation support from healthcare professionals.

## 4 Outcomes and measures

### 4.1 Blood pressure (BP), heart rate variability (HRV), and arterial stiffness

Office BP is measured following a one-hour rest in accordance with adult (Williams et al., 2018) and pediatric (Flynn et al., 2017) guidelines using the Omron HBP-1320 blood pressure monitor device. This is performed for mothers, fathers, and children. 24-hour BP monitoring is performed in mothers and children in accordance with adult (Parati et al., 2014) and pediatric (Flynn et al., 2014) guidelines using the Schiller BR-102 plus device. Profiling of autonomic nervous system function is performed during the study visit by recording Heart Rate Variability (HRV) during a 10 min Holter monitoring at rest in mothers and children using the Bittium Faros™ 360 device (Bittium Ltd). Arterial stiffness is assessed with tonometry (Complior Analyse) for mothers and children.

### 4.2 Questionnaires and dietary intake

The following information will be collected from parents by questionnaires: stress (Perceived Stress Scale, PSS) (Järvelä-Reijonen et al., 2016), quality of life, medical history, use of medication, socioeconomic status (education, income, employment), sleep, use of tobacco and alcohol, and a family history of CVDs. A questionnaire including questions on the awareness of PE related later life morbidity is provided to mothers and fathers. Symptoms of depression in children are assessed via a self-reported questionnaire, the Children’s Depression Inventory, CDI (Kovacs, 1992). Validated and updated food frequency questionnaires (FFQ) are used to assess dietary intake (Erkkola et al., 2001). The following background information is collected from children: medical history, use of medication, physical activity and sleep. A validated FFQ particularly developed for children and modified to capture consumption patterns of fats, cereal products, and salt rich foods is used to assess quality of diet (Korkalo et al., 2019).

### 4.3 Physical activity

Both subjective and objective data is collected on physical activity and sedentary behaviour. Subjective information is based on questions in the International Physical Activity Questionnaire (IPAQ), which is validated in the general population in 12 countries including Finland (Craig et al., 2003). Objective information is based on accelerometer (RM-42, UKK Terveyspalvelut Oy, Tampere Finland), which has proved reliable in assessing physical activity in adults (Vähä‐Ypyä et al., 2015) and youth (Aittasalo et al., 2015). In children, accelerometers are used only as questionnaires are not recommended in young children less than 10 years of age (Dollman et al., 2009), and parental proxy-reports have been shown unreliable (Corder et al., 2008).

### 4.4 Laboratory measurements

Cardiovascular and metabolic risk profiles are assessed from mothers, fathers and children by venous blood samples taken after an overnight fast for analysis of serum lipids and lipoproteins, insulin, glucose and inflammatory marker hs-CRP. Morning urine samples are collected from mothers only to assess microalbumin creatinine ratio. Serum and plasma samples are stored for other relevant biomarker analyses (e.g. myocardial markers, brain natriuretic peptide (BNP), anti-angiogenic marker soluble fms-like tyrosine kinase (sFlt-1), fatty acid binding protein 4 (FABP4), leptin, resistin, adiponectin, growth/differentiation factor 15 (GDF15).

### 4.5 Body anthropometrics and composition

Height and weight are measured with a Seca 285 scale and stadiometer (Seca GmbH, Hamburg, Germany). Waist circumference and other anthropometric measures of body dimensions are measured with a tape measure to the closest millimeter. Body composition is assessed with bioimpedance analysis using the InBody 720 device. All these measures are performed in mothers, fathers and children.

### 4.6 Cardiovascular imaging

Subclinical atherosclerosis in mothers and vascular health in children will be assessed with carotid, brachial and radial artery arterial wall layer quantification and plaque assessments using Vevo MD (Visualsonics) and Vivid 7 (GE) (Sarkola et al., 2010, Sundholm et al., 2019, Touboul et al., 2012). Left ventricular mass, systolic and diastolic function will be determined in mothers and children via ultrasound Vivid 7/E9, EchoPac) (Lopez et al., 2010).

### 4.7 Cardiovascular risk scores

Since the CVD risk profile might be low in this relatively young population, previously established and published scoring tools will be used in order to provide an estimate of risk for future CVD events (i.e., heart attack, stroke, and coronary artery revascularization). These include 10-year risk scores, Framingham (D’Agostino et al., 2008) and Reynolds (Ridker et al., 2007), 30-year risk scores (Pencina et al., 2009) and lifetime risk scores (Lloyd-Jones et al., 2006). In the current study, we will calculate individual (extrapolated) 10-and 30-year cardiovascular event risks using these four different risk prediction models. However, these risk prediction models do not account for obstetric history.

All measurements and timing in the participating family members are summarized in Supplementary Table 1.

### 4.8 Data analyses, outcomes and process evaluation

Primary outcome of the intervention for mothers and children is mean 24-hour BP change (baseline − 12 months). Secondary outcomes in mothers and children include change in BP 24-hour variability, arterial stiffness (pulse wave velocity), heart rate variability, adiposity, dietary intake, physical and sedentary behavior, smoking (mothers), laboratory measurements of lipids, glucose and inflammation. Arterial layer thickness, left ventricular mass, systolic and diastolic function is, in addition to the above mentioned parameters, compared between PE and non-PE mothers and children at baseline. The FINNCARE study outcome variables are listed on ClinicalTrials.gov (NCT04676295). Primary and secondary outcome variables will be assessed with ANCOVAs or GLM-modelling adjusting for differences at baseline as well as including important confounders in the analyses.

Process evaluation (Table 2) will examine intervention feasibility and it will follow Medical Research Council (MRC) guidance on process evaluations for complex interventions (Moore et al., 2015).

**Table 2:** Evaluation of the lifestyle intervention.

Evaluation component	Indicator(s)	Measure(s)
*Effectiveness, primary outcome*		
Cardiovascular health	Blood pressure	Blood pressure measurements (office and 24 h) at 12 months

*Effectiveness, secondary outcomes*		
Diet	Quality of fat (parents and children) Consumption of foods rich in fiber (parents and children) Use of salt (parents and children) Meeting self-set goals (parents)	Food frequency questionnaire (FFQ) at baseline and 12 months FFQ at baseline and 12 months FFQ at baseline and 12 months Data accumulated in the web portal at 12 months
Physical activity	Daily steps (parents and children) Daily stationary behavior (parents and children) Weekly number of sessions and minutes of overall and leisure physical activity (parents) Meeting self-set goals (parents and children)	UKK-RM42 accelerometer at baseline and 12 months Questionnaire at baseline and 12 months Data accumulated in the web portal at 12 months
Smoking	Proportion of smokers (parents)	Questionnaire at baseline and 12 months

*Process evaluation*		
Reach	Participation rate of the families recruited (family) Representativeness of the families participating: parents’ and children’s age, socio-economics	Research database on study visits Questionnaire at baseline
Compliance	Participation rate in measurements (parents and children separately) Proportion of sessions completed in the web-based portal (parents and children separately)	Number of study visits and questionnaires completed at baseline and 12 months Data accumulated in the web portal at 12 months
Acceptability	Usefulness, ease of use, credibility and satisfaction of the web-based portal, occurrence of technical problems (parents and children)	Questionnaire at 12 months Proportion of web portal sessions completed by the families

### 4.9 Future follow-up and registry linkages

Following the 12-month assessment the study cohort will be followed up by national register linkages (first linkage estimated in 2024–25). Data from national healthcare registers will allow assessment of key covariates and follow-up of outcomes long-term after the 12-month follow-up. Maternal and paternal CVD and comorbidities will be assessed through 1) Care Register for Health Care showing 10th revision of the International Classification of Diseases (ICD-10) codes for all inpatient and outpatient treatments in specialty care and 2) Medication data from National Social Insurance Institution purchases of prescribed medications and medication special reimbursement. Grandparental body data from these registers allow adjustment for family history. In children, outcome body mass index (BMI) is available from the national primary care register from ∼2011 onwards (typically measured once a year between infancy and ∼16 y).

## 5 Ethical aspects

The approval of the study protocol is granted (HUS/3347/2018) from the Ethics Committee of the Hospital District of Helsinki and Uusimaa in December 2018. Informed consent is required for study participation.

## 6 Implications

Current study performs detailed phenotyping and evaluates first time the impact of PE on long-term CVD risk and CVD progression not only in women with a history of PE but also in their children and partners. FINNCARE provides needed evidence whether and how a long-term lifestyle intervention could improve cardiovascular health and CVD risk in PE families. The interactive web-based portal approach to deliver the lifestyle intervention by professionals could potentially be modified to primary health care needs of early CVD prevention in targeted CVD risk groups including PE families.

## Authors contributions

TJ is the principal investigator and the coordinator on the current trial. HL is the study chair and TS the study director. TJ, HL, TS and SH designed the study. MA provided expertise concerning design and evaluation of lifestyle intervention trial. AK and MR are PhD-students involved in the recruitment of participants, intervention, data collection and management. TJ drafted the first version of the manuscript and wrote the final version together with TS. All other authors were responsible for revising the manuscript. All authors approved the final version of the manuscript.

## Funding

Study is supported by Juho Vainio Foundation (TJ), Jane and Aatos Erkko Foundation (HL), Päivikki and Sakari Sohlberg Foundation (HL), Research Funds of the University of Helsinki, Government special subsidy for health sciences (In Finnish; Valtion tutkimusrahoitus) at the Hospital District of Helsinki and Uusimaa (SH), Sigrid Juselius Foundation (TS), The Medical Society of Finland (HL, TS, MR), Medicinska understödsföreningen Liv och Hälsa rf (TS), Finnish Foundation for Pediatric Research (TS), and Dorothea Olivia, Karl Walter och Jarl Walter Perklén foundation (TS, MR)**,** the Competitive State Research Financing of the Expert Responsibility area of Tampere University Hospital (HL).

## Competing interests

None declared.
