# Supplementary material for: Protocol: A randomized controlled trial to assess effectiveness of a 12-month lifestyle intervention to reduce cardiovascular disease risk in families ten years after pre-eclampsia (FINNCARE)
Source: Prev Med Rep. 2022 Feb 8;26:101731. doi: 10.1016/j.pmedr.2022.101731 (PMC8861388; doi:10.1016/j.pmedr.2022.101731)
Supplement: Supplementary data 1 [file mmc1.docx]

**Supplementary table 1.** Measurements and timing in the participating family members.

|  | **Baseline** | | | **12 month follow-up** | | |
| --- | --- | --- | --- | --- | --- | --- |
|  | Mother | Child | Father | Mother | Child | Father |
| Background information/ questionnaires | ● | ● | ● | ● | ● | ● |
| Food frequency questionnaires (FFQ) | ● | ● | ● | ● | ● | ● |
| Body anthropometrics and composition | ● | ● | ● | ● | ● | ● |
| Blood sample | ● | ● | ● | ● | ● | ● |
| Urine sample | ● |  |  | ● |  |  |
| Office blood pressure | ● | ● | ● | ● | ● | ● |
| 24h blood pressure monitoring | ● | ● |  | ● | ● |  |
| Heart rate variability (HRV) | ● | ● |  | ● | ● |  |
| Cardiovascular imaging | ● | ● |  | ● | ● |  |
| Pulse wave velocity | ● | ● |  | ● | ● |  |
| Accelometer (7 days) | ● | ● |  | ● | ● |  |
